# Telemedicine in patients with obsessive–compulsive disorder after deep brain stimulation: a case series

**DOI:** 10.3389/fnhum.2024.1296726

**Published:** 2024-02-14

**Authors:** Xiaonan Wan, Zhengyu Lin, Zhitong Zeng, Yingying Zhang, Chengcheng Duan, Chencheng Zhang, Dianyou Li

**Affiliations:** ^1^Department of Neurosurgery, Center for Functional Neurosurgery, Ruijin Hospital, Shanghai Jiao Tong University School of Medicine, Shanghai, China; ^2^Institute of Science and Technology for Brain-Inspired Intelligence, Fudan University, Shanghai, China; ^3^Clinical Neuroscience Center, Ruijin Hospital Luwan Branch, Shanghai Jiao Tong University School of Medicine, Shanghai, China

**Keywords:** obsessive-compulsive disorder, deep brain stimulation, telemedicine, remote programming, COVID-19

## Abstract

**Background:**

Patients suffering from refractory obsessive-compulsive disorder (OCD) who have undergone deep brain stimulation (DBS) surgery require repeated in-person programming visits. These sessions could be labor-intensive and may not always be feasible, particularly when in-person hospital visits are restricted. Telemedicine is emerging as a potential supplementary tool for post-operative care. However, its reliability and feasibility still require further validation due to the unconventional methods of interaction.

**Methods:**

A study was conducted on three patients with refractory OCD who had undergone DBS. Most of their programming sessions were completed via a remote programming system. These patients were recruited and monitored for a year. Changes in their clinical symptoms were assessed using the Yale-Brown Obsessive-Compulsive Scale–Second Edition (Y-BOCS-II), the Hamilton Anxiety Scale-14 (HAMA), the Hamilton Depression Scale-17 (HAMD), and the Short Form 36 Health Survey Questionnaire (SF-36). The scores from these assessments were reported.

**Results:**

At the last follow-up, two out of three patients were identified as responders, with their Y-BOCS-II scores improving by more than 35% (P1: 51%, P3: 42%). These patients also experienced some mood benefits. All patients observed a decrease in travel expenses during the study period. No severe adverse events were reported throughout the study.

**Conclusion:**

The group of patients showed improvement in their OCD symptoms within a 1-year follow-up period after DBS surgery, without compromising safety or benefits. This suggests that telemedicine could be a valuable supplementary tool when in-person visits are limited.

## Introduction

Over 10% of patients with obsessive-compulsive disorder (OCD) are resistant to first-line psychopharmacological treatment, either alone or in combination with cognitive-behavior therapy (CBT), which includes exposure and response prevention (Denys, 2006). For those with severe, treatment-resistant symptoms, deep brain stimulation (DBS) has been granted a Humanitarian Device Exemption (HDE) by the US Food and Drug Administration (FDA) (U.S. Food and Drug Administration, 2009). DBS has demonstrated cost-effectiveness (Ooms et al., 2017; Gadot et al., 2022), although its efficacy partially depends on the quality of post-operative management (van Westen et al., 2021). Conventional DBS programming sessions necessitate repeated in-person follow-ups at specialized centers, posing a significant burden on both patients and their caregivers, particularly those with disabilities or those living far from such centers (Pintér et al., 2022).

Telemedicine, which includes teleconsultation, telemonitoring, and telerehabilitation, has been rapidly adopted by healthcare professionals to provide remote medical service. Specifically, the remote programming system of DBS involves parameter adjustment, battery status check, and device troubleshooting (Zhang et al., 2018). Several studies have validated the cost-effectiveness and satisfaction of telemedicine among patients with movement disorders (Esper et al., 2022; Nie et al., 2022). However, studies focusing on the use of DBS telemedicine in psychiatric disorders are relatively scarce and mostly involve short-term follow-up (<6 months) (Lin et al., 2020).

In this study, we retrospectively evaluated the 1-year outcomes of three OCD patients who underwent bilateral Bed Nucleus of the Stria Terminalis (BNST) DBS. This work aims to contribute to a better understanding of post-operative management through telemedicine for OCD patients with DBS.

## Methods

### Participants

The study involved three refractory OCD patients who had undergone bilateral BNST DBS and finished 1-year post-operative programming sessions partially via DBS telemedicine. All patients were diagnosed by an experienced psychiatrist using a standardized mental health examination based on the *Diagnostic and Statistical Manual of Mental Disorders, Fifth Edition (DSM-5)* (Association Psychiatric Association, 2013).

The surgical procedure and criteria were similar to those used in our previous lesion surgery study (Yin et al., 2018; Zhang et al., 2021a). Non-directional electrodes (contact length = 1.5 mm, inter-electrode spacing = 1.5 mm, Model 1200, SceneRay, China) and neurostimulators capable of remote programming (Model 1180, SceneRay, China) were implanted (Li et al., 2017). The neurostimulators were activated for temporary stimulation before discharge. Written informed consent for the surgery and subsequent follow-ups was obtained prior to surgery. The study protocol was reviewed by the local Institutional Review Board and complied with the *Declaration of Helsinki*.

### Remote programming

Similar to conventional in-person programming sessions, doctors initially collected clinical symptom information and medication specifics from patients or their families, then informed them of potential side effects related to stimulation. After the IPG was connected, programmers checked the impedance and verify the position of the electrodes using fused images from post-operative CT and preoperative MRI scans.

Each contact was examined using monopolar stimulation, applied in increments of 0.5–1 V up to around 5 V (with a fixed pulse width of 180 μs and frequency of 130 Hz). Acute clinical effects (such as decreased anxiety, less intrusive obsessions, increased spontaneous speech, etc.) and stimulation-related side effects (like dizziness, irritability, blushing, palpitation, etc.) for each parameter were recorded. These acute effects were observed by programmers via remote video conferencing and used to determine the therapeutic window for each contact. Optimal or sub-optimal parameters were set as different groups (van Westen et al., 2021).

Long-term clinical responses (like reduction of obsessive thoughts and compulsive behaviors) and side effects (such as sleep problems, hypomania, etc.) could take days to weeks to manifest and were difficult to predict during the programming process. We recommended patients to receive programming once every 1 to 2 weeks for timely adjustment. However, the actual programming interval varied among patients, who usually scheduled programming appointments based on their own feelings. The product used in this study could retain up to 16 parameter groups. Once a new group was set, the previously used parameter was saved in case the newer one was not tolerated. Psychotropic medications remained unchanged for 1 month after each programming session and were adjusted by the psychiatrist based on clinical assessment at each follow-up visit.

### Cyber security

Both patients and programmers were required to log on to the remote programming system with a secured username and password. The data generated during the programming session was stored in a secured cloud-based database, ensuring the privacy and security of patient information (Chen et al., 2014; Zhang et al., 2020).

### Data collection

Participants were assessed at 1 week before surgery (baseline), and at 1-, 3-, 6-, and 12-month post-operative follow-ups, with the 12-month follow-up being the last (LFU). The primary outcome was the change in scores of the Yale-Brown Obsessive-Compulsive Scale–Second Edition (Y-BOCS-II, Mandarin Chinese Version) (Zhang et al., 2019). Patients with an improvement of more than 35% compared with baseline were classified as responders. Other outcomes were assessed on the change in scores of the Short Form 36 Health Survey Questionnaire (SF-36) (Brazier et al., 1992), the Hamilton Depression Scale-17 (HAMD) (Hamilton, 1960) and the Hamilton Anxiety Scale-14 (HAMA) (Hamilton, 1959), with higher scores representing better Quality of Life (QoL), greater depression, and anxiety severity, respectively. Medication adjustment and adverse events were also documented in the study.

Additionally, we estimated the approximate cost of post-operative management for each patient based on the distance between their residences and our center. Cost components included: (a) transportation (focusing on train and taxi fares); and (b) registration fee for in-person programming. The fee for remote programming was 200 Yuan (Chinese Yuan) per session.

Due to the small size of this study, a descriptive analysis of outcome was performed. Data were collected using Excel (Microsoft, San Jose, CA, USA).

## Results

(Table 1) presents the characteristics and clinical improvements of each patient within the study time frame. One patient (P1) had a comorbid Major Depressive Disorder (MDD) according to the diagnostic criteria of DSM-5 (Association Psychiatric Association, 2013).

**TABLE 1:** Characteristics and clinical outcomes of enrolled patients.

Case	Diagnosis	Age at surgery	Duration of disease	Baseline/LFU				
**No.**		**(Years)**	**(Years)**	**HAMD**	**HAMA**	**Y-BOCS II**	**SF-36**	**Medication**
P1	OCD and MDD	30	7	17/1	14/4	41/20	127/400	Flu 250 mg, Que 20 mg/Flu 200 mg
P2	OCD	31	5	10/10	16/10	35/27	442/414	Ser 200 mg/Ser 100 mg
P3	OCD	29	7	13/0	12/0	26/15	608/788	Flu 100 mg/Flu 100 mg

LFU, last follow-up; Flu, fluvoxamine; Que, Quetiapine; Ser, sertraline.

At the LFU, two patients (P1, P3) showed significant improvement in OCD symptoms (51.2, 42.3% score reduction on the Y-BOCS-II), compared with the baseline. They also demonstrated marked improvement in depression and anxiety (with a reduction of 94.1, 100% on the HAMD score and 76.5, 100% on the HAMA score). One patient (P2) showed the most modest benefit in OCD symptom (22.9% on the Y-BOCS-II) and was deemed as non-responder, along with a modest improvement in symptoms of depression and anxiety (with a reduction of 0 and 37.5% on the HAMD and HAMA score).

**FIGURE 1:**
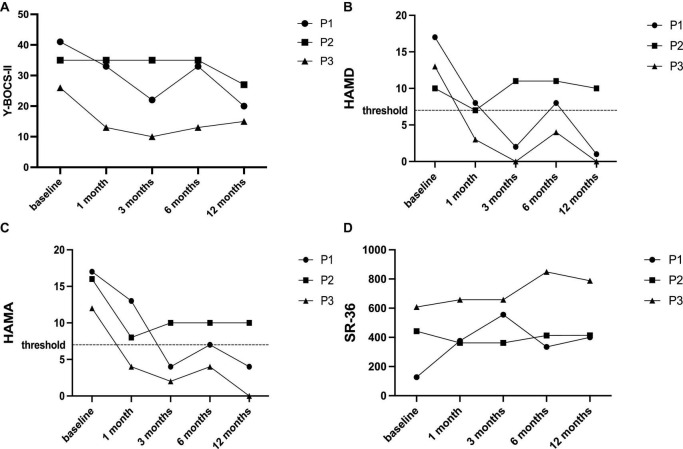
Changes of main outcomes at baseline and each follow-up. **(A)** YBOCS-II score; **(B)** HAMD score; **(C)** HAMA score; and **(D)** SF-36 score.

At the 3-month follow-up, 2 patients (P1, P3) were classified as responders. However, at the 6-month follow-up, an aggravation of OCD symptoms was observed in them, and P1 became a non-responder, while P3 sustained relatively considerable improvement. At the LFU, improvement in Y-BOCS-II score was noted in all patients, but P2 was still classified as a non-responder (Figure 1A).

In terms of mood, the changing trends of HAMD and HAMA over the timeframe were consistent. Improvements in depression and anxiety were concurrently observed in two responders (P1, P3) at LFU but fluctuated at 6-month follow-up (Figures 1B, C). Similarly, these responders obtained improvement in QoL at the last follow-up compared to the baseline (Figure 1D).

A total of 40 programming sessions were completed before the LFU, 38 of which were completed remotely (Table 2). The average cost of each in-person programming session for the 3 patients was 208 Yuan, 1322 Yuan and 444 Yuan, respectively, which was higher than the fee of each telemedicine procedure.

**TABLE 2:** Programming sessions and cost estimated for each patient.

Case	Distance (km)	Programming frequency			Cost estimated (Yuan)		
**(No)**		**Telemedicine**	**In-person**	**Total**	**Telemedicine**	**In-person**	**Total**
P1	114	19	0	19	3800	0	3800
P2	1679	17	1	18	3400	1322	4722
P3	340	2	1	3	400	444	844

With respect to adverse events, P2 reported a headache which could be ameliorated after parameter adjustment (reducing stimulation intensity), acute depressive symptoms that lasted for 3 days after drug withdrawal at the 3-month follow-up, and nocturne dysthymia at the LFU (ameliorated within 5 days after switching to the former parameter group). No severe adverse events were observed.

## Discussion

This study highlights the utility of DBS telemedicine in the post-operative management of OCD patients. This approach allowed follow-up sessions to continue and patients to gain clinical benefits during the COVID-19 epidemic when visits were constrained.

Patient P1 exhibited a response in the 3-month and final follow-ups, but not in the 6-month follow-up (Figure 1A). This fluctuation of symptom was also documented in previous studies (Fayad et al., 2016; Graat et al., 2021), probably due to daily life stress or other factors. P2 was consistently classified as a non-responder throughout the study, which is not unusual (Gadot et al., 2022). Factors such as age of disease onset, sexual/religious symptoms, and target selection may be potential predictors of response (Guzick et al., 2020). However, a recent large-sample real-world study found no significant predictors of response (Mar-Barrutia et al., 2022).

Three patients completed 40 programming sessions before LFU, with 38 conducted remotely. Considering the distance between these patients, telemedicine significantly reduced travel cost during post-operative management. In this study, we estimated the approximate cost primarily by the fare of trips, the actual cost of in-person follow-ups could be higher considering the loss of working time of both patients and their caregivers.

Two patients (P1 and P2), who maintained a relatively high level of depression and anxiety, requested more remote programming sessions. Increased programming requests are often observed in patients with anxiety or depression in clinical practice. We hypothesized that remote programming could provide psychological support to some extent. In a previous study (Zhang et al., 2021b), some patients expressed satisfaction with the remote programming even without any actual adjustment. Patient P3 chose remote programming in the last two sessions after obtaining satisfactory improvement. An increase in the number of programming sessions was observed after the lock-down measures of COVID-19 (Zhang et al., 2021b), suggesting that as patients become more accustomed to telemedicine, demand for remote programming may rise.

In our experience, remote programming might be more recommended for those with OCD compared to movement disorders. Programming for patients with movement disorders primarily relies on the evaluation of motor performance (Picillo et al., 2016), which is more challenging to observe in a teleconferencing setting (Lin et al., 2020). Furthermore, the impact of voltage increments on mental disorders is not always immediate, necessitating more frequent adjustments (van Westen et al., 2021).

The results of this case series are promising but should be considered preliminary due to the following limitations. The study included only a small patient sample without a comparison group, and there was potential for bias and confounding. Additionally, this study was conducted during the COVID-19 period, and travel restrictions affected the patients’ choice of programming method. Further research is needed to support or refute these findings before drawing any firm conclusions.

## Conclusion

Repeated in-person visits are burdensome for OCD patients who have undergone DBS. The use of DBS telemedicine proves beneficial for the post-operative management of these patients. However, further exploration is needed to establish participant selection criteria and reach a consensus on the programming algorithm.

## Data availability statement

The raw data supporting the conclusions of this article will be made available by the authors, without undue reservation.

## Ethics statement

The studies involving humans were approved by Ethics Committee of Ruijin Hospital. The studies were conducted in accordance with the local legislation and institutional requirements. The participants provided their written informed consent to participate in this study. Written informed consent was obtained from the individual(s) for the publication of any potentially identifiable images or data included in this article.

## Author contributions

XW: Writing – original draft. ZL: Formal Analysis, Writing – review and editing. ZZ: Writing – review and editing. YZ: Data curation, Writing – review and editing. CD: Writing – review and editing. CZ: Conceptualization, Writing – review and editing. DL: Conceptualization, Supervision, Writing – review and editing.
